# Sustainable Leadership, Workplace Integration, and Resilience Among Staff Nurses: A Cross-Sectional Correlational Study

**DOI:** 10.3390/healthcare14162537

**Published:** 2026-08-13

**Authors:** Eman Ramadan Abdalfadeel, Eman Kamel Hossny, Ahmed Zinhom Elkady, Intisar Alsheikh Mohamed, Ishraga Abdelgadir Ibrahim Mohamed, Eman Yosry Mohamed

**Affiliations:** 1 Primary Care Nursing Department, Faculty of Nursing, Al-Ahliyya Amman University, Amman 19328, Jordan; 2 Nursing Administration Department, Faculty of Nursing, Cairo University, Giza 12613, Egypt; emanhout@gmail.com; 3 Community and Mental Health Nursing Department, Faculty of Nursing, Zarqa University, Zarqa 13132, Jordan; 4 Nursing Administration Department, Faculty of Nursing, Assiut University, Assiut 71515, Egypt; 5 Nursing Department, North Private College of Nursing, Arar 73311, Saudi Arabia; ahmadzinhom@nec.edu.sa; 6 Department of Nursing, College of Applied Medical Sciences, University of Jeddah, Jeddah 21589, Saudi Arabia; iaalsheikh@uj.edu.sa; 7 Critical Care Nursing, Department of Nursing, College of Applied Medical Sciences, University of Jeddah, Jeddah 21589, Saudi Arabia; imohamed@uj.edu.sa

**Keywords:** sustainable leadership, workplace integration, resilience, nursing workforce, healthcare management

## Abstract

**Background**: Sustainable leadership has emerged as a critical approach for promoting long-term organizational effectiveness and workforce well-being in healthcare settings. However, empirical evidence linking sustainable leadership to workplace integration and resilience among nurses remains limited. **Objective**: This study aimed to examine the association between sustainable leadership and both workplace integration and resilience among staff nurses. **Methods**: A descriptive cross-sectional correlational design was used. Data were collected from 200 staff nurses working in intensive and intermediate care units at a tertiary healthcare institution. Standardized instruments were used to measure sustainable leadership, workplace integration, and resilience. Data were analyzed using descriptive statistics, Pearson correlation, and linear regression analysis. **Results**: Sustainable leadership was positively associated with workplace integration (r = 0.43, *p* < 0.001) and resilience (r = 0.24, *p* < 0.001). Regression analysis indicated that sustainable leadership was associated with workplace integration (β = 0.43, R^2^ = 0.18, *p* < 0.001) and resilience (β = 0.24, R^2^ = 0.10, *p* < 0.001), explaining a modest proportion of variance in both outcomes. **Conclusions**: Sustainable leadership is significantly associated with improved workplace integration and resilience among nurses. Healthcare organizations should adopt sustainable leadership practices such as providing consistent mentorship and professional development chances to improve workplace integration, and implementing supportive supervision and wellness initiatives to improve nurse resilience. However, the relatively low explained variance suggests that additional individual and organizational factors contribute to these outcomes. Therefore, sustainable leadership should be integrated with complementary strategies, including peer support programs and workload management interventions, to comprehensively strengthen workforce integration and resilience.

## 1. Introduction

Healthcare systems are increasingly challenged by workforce shortages, high job demands, and the need to maintain quality care under complex and rapidly changing conditions [1,2]. Nurses, as the largest group of healthcare professionals, are particularly exposed to these pressures, which may negatively affect their well-being, performance, and retention [3]. Therefore, identifying organizational factors that support nurses’ adaptation, integration, and psychological resilience has become a priority for healthcare management [4].

Leadership is a key determinant of workplace environment and staff outcomes in healthcare settings [5]. Traditional leadership approaches that focus primarily on short-term performance may be insufficient to address current healthcare challenges [6]. In contrast, sustainable leadership has emerged as a more comprehensive approach that emphasizes long-term thinking, ethical decision-making, employee development, and organizational responsibility [7,8]. This leadership style aims to balance organizational performance with staff well-being and system sustainability.

Sustainable leadership is characterized by practices such as stakeholder engagement, empowerment, accountability, and capacity building [8,9]. These practices can contribute to creating supportive work environments that enhance collaboration, trust, and professional development among nurses. Such environments are essential for promoting workplace integration, defined as the extent to which employees feel connected, supported, and aligned with organizational values [10].

Workplace integration plays a critical role in improving job satisfaction, reducing turnover, and enhancing team performance [11]. Nurses who are well integrated into their work environment are more likely to engage in collaborative practices and demonstrate organizational commitment [12]. In addition, workplace integration may act as a protective factor against occupational stress, thereby supporting resilience [13].

Resilience, defined as the ability to adapt and maintain functioning in the face of stress and adversity, is essential for nurses working in high-pressure clinical environments [14]. Resilient nurses are better able to cope with workload demands, recover from challenges, and sustain performance [15]. Although leadership has been suggested as an important factor influencing resilience, the mechanisms through which leadership contributes to resilience remain insufficiently explored [16].

Previous research has indicated that positive leadership styles are associated with improved staff outcomes, including well-being, engagement, performance, work motivation, and occupational mental health [17]. However, empirical evidence specifically examining the role of sustainable leadership in shaping workplace integration and resilience among nurses is still limited, particularly in developing healthcare contexts [18]. Furthermore, most existing studies focus on single outcomes rather than examining the interrelated nature of leadership, integration, and resilience.

The proposed conceptual framework for this study assumes that sustainable leadership acts as a critical organizational resource that shapes nurses’ workplace experiences and adaptive capacities. Sustainable leadership emphasizes long-term employee well-being, ethical decision-making, empowerment, and the creation of supportive work environments that promote both organizational sustainability and employee development. Within the Job Demands–Resources (JD-R) framework, leadership behaviors represent essential job resources that help employees cope with workplace demands and foster positive work outcomes. Recent evidence indicates that effective nursing leadership is strongly associated with improved work environments, enhanced professional relationships, and better staff outcomes, all of which contribute to nurses’ capacity to adapt to workplace challenges and maintain resilience. In addition, contemporary leadership approaches such as organizational agility and ambidextrous leadership have been shown to significantly enhance nurses’ readiness for change, further strengthening their adaptability and responsiveness within dynamic healthcare systems [19].

Furthermore, the framework posits that workplace integration serves as an important mechanism through which sustainable leadership promotes resilience. Workplace integration reflects nurses’ sense of belonging, professional acceptance, collaboration, and social connectedness within the organization. According to Conservation of Resources (COR) theory, supportive interpersonal relationships and organizational resources protect individuals from stress and facilitate adaptation to adversity. Contemporary resilience literature emphasizes that resilience is a dynamic process arising from the interaction between individual capabilities and environmental resources rather than a fixed personal trait [20]. Similarly, multilevel resilience research demonstrates that supportive work environments and positive team interactions are fundamental antecedents of workplace resilience [21]. Therefore, this conceptual framework proposes that sustainable leadership fosters greater workplace integration, which subsequently enhances resilience among staff nurses.

Addressing this gap is important for developing evidence-based leadership strategies that support healthcare workforce sustainability. Understanding how sustainable leadership is associated with both workplace integration and resilience may provide insights into improving organizational practices and staff outcomes.

Therefore, this study aimed to examine the association between sustainable leadership and workplace integration and resilience among staff nurses in clinical settings.

Research Hypotheses:

**H1.**  *Sustainable leadership is positively associated with workplace integration among staff nurses.*

**H2.**  *Sustainable leadership is positively associated with resilience among staff nurses.*

## 2. Materials and Methods

### 2.1. Research Design

A descriptive cross-sectional correlational design was employed to examine the association between sustainable leadership, workplace integration, and resilience among staff nurses.

### 2.2. Setting

The study was conducted in the intensive care units (ICUs) and intermediate care units of the National Heart Institute, Cairo University, it is a major tertiary referral center for cardiac care in Egypt. These units were purposefully selected because their high-acuity, and team-dependent environment provides an optimal context to examine the relationship between sustainable leadership, workplace integration, and resilience among staff nurses. In such settings, as nurses frequently encounter emotional exhaustion, ethical dilemmas, and rapid clinical deterioration. Moreover, workplace integration is continuously challenged by high patient turnover, and interdisciplinary teamwork, making these units highly relevant to our research model. The institute’s affiliation with Cairo University facilitated ethical approval, administrative support, and access to a stable nursing workforce, further justifying the choice of this context.

### 2.3. Participants

The required sample size was estimated using G*Power software (Düsseldorf, Germany: Heinrich-Heine-Universität Düsseldorf, 2009, version 3.1.9.7), The calculation was based on a two-tailed correlation assuming an effect size of 0.30, a statistical power of 1−β=0.80, and a significance level of α = 0.05. The minimum required sample size was 100 participants. This effect size was chosen based on Cohen [22] conventions and is consistent with previous studies in this research area. To enhance the robustness of the analysis, a total of 200 nurses were recruited using convenience sampling, all of them were eligible for this study. Of the 240 nurses invited to participate, 200 agreed to take part, yielding a response rate of 83.3%. No incentives were given for participation.

### 2.4. Data Collection Procedures

Data was collected using a structured, self-administered questionnaire distributed electronically via a secure online link. Participants received information about the study purpose, voluntary participation, and confidentiality before completing the questionnaire. The estimated time for completion was 15–20 min. Reminders were sent to improve response rates. Data was collected between February and March 2026.

### 2.5. Study Instruments

Three standardized and validated instruments were used to measure sustainable leadership, workplace integration, and resilience among staff nurses.

#### 2.5.1. Sustainable Leadership Questionnaire

Sustainable leadership was assessed using the Sustainable Leadership Questionnaire developed by Avery and Bergsteiner [23]. The instrument consists of 16 items evaluating key dimensions of sustainable leadership, including ethical decision-making, stakeholder engagement, accountability, long-term orientation, and capacity building. Participants responded using a five-point Likert scale (1 = strongly disagree, 5 = strongly agree). Higher scores indicate greater perceived sustainable leadership. Previous research reported good psychometric properties, with Cronbach’s alpha values exceeding 0.80.

#### 2.5.2. Workplace Integration Questionnaire

Workplace integration was measured using the instrument developed by Xu et al. [24]. This questionnaire comprises five dimensions: communication and collaboration, teamwork, organizational belonging, support and inclusion, and adaptation and engagement. Responses were rated on a five-point Likert scale (1 = strongly disagree, 5 = strongly agree). Higher scores reflect stronger workplace integration and a greater sense of inclusion. The instrument has shown good reliability and validity in healthcare settings.

#### 2.5.3. Resilience Assessment Questionnaire

Resilience was measured using the 15-item version of the Connor–Davidson Resilience Scale (CD-RISC-15), developed by Connor and Davidson [25]. The scale was structured around five resilience dimensions: Emotional Stability (3 items), Adaptability (3 items), Coping Strategies (3 items), Optimism and Positive Outlook (3 items), and Professional Endurance (3 items). Each item was rated on a three-point Likert scale (1 = Disagree, 2 = Neutral, 3 = Agree). The total score was calculated by summing responses, with higher scores indicating greater resilience. Domain scores were also calculated to pinpoint specific strengths and areas for growth.

Content validity of the custom resilience scale was established through a panel of seven experts in nursing and healthcare management. The experts independently rated each item’s relevance and clarity using a four-point scale. Based on their ratings, the Scale Content Validity Index (S-CVI) was 0.91, indicating excellent content validity. Reliability was assessed using Cronbach’s alpha, which yielded 0.87 for the overall scale, indicating good internal consistency.

### 2.6. Validity and Reliability

Content validity was established through expert review by a panel of seven specialists, including three professors of nursing administration, two healthcare management experts, and two senior nursing researchers with psychometric expertise. The panel independently rated items, and S-CVI values were calculated for each instrument: 0.88 for the Sustainable Leadership Questionnaire, 0.83 for the Workplace Integration Questionnaire, and 0.91 for the Resilience Assessment Questionnaire, all indicating acceptable to excellent content validity. Reliability was determined using Cronbach’s alpha: 0.81 for the Sustainable Leadership Questionnaire, 0.90 for the Workplace Integration Questionnaire.

### 2.7. Data Analysis

Data were analyzed using the Statistical Package for the Social Sciences (SPSS) version 30.0 (Armonk, NY, USA: IBM Corp., 2025. Descriptive statistics (frequencies, means, and standard deviations) were used to summarize participants’ characteristics and study variables. Pearson’s correlation coefficient was used to examine relationships between variables. Linear regression analysis was conducted to examine the statistical association between sustainable leadership and workplace integration and resilience. Statistical significance was set at *p* ≤ 0.05.

### 2.8. Ethical Considerations

The study was conducted in accordance with the Declaration of Helsinki. Ethical approval was obtained from the Institutional Review Board of the Faculty of Nursing, Cairo University (Approval No. RHDIRB2019041701) (Date: 11 January 2026). Participation was voluntary, and informed consent was obtained from all participants prior to data collection. Confidentiality and anonymity were ensured throughout the study.

### 2.9. Methodological Considerations

Given that all data were collected using self-report questionnaires at a single time point, the study may be susceptible to common method bias (CMB). To address this concern, several procedural and statistical remedies were implemented.

Procedural remedies: Participants were assured of the anonymity and confidentiality of their responses to minimize social desirability bias. The questionnaire items were reviewed for clarity and simplicity to avoid unclear, vague or complex wording. Additionally, the independent and dependent variables were presented in separate sections of the questionnaire, and the order of items within each section was counter-balanced as possible to reduce response consistency biases.

To assess common method bias, Harman’s single-factor test was performed. The unrotated factor solution showed that the first factor accounted for 28.5% of the total variance, below the 50% threshold (Podsakoff et al [26]. indicating that common method bias was not a major concern.

## 3. Results

The results of Table 1 showed that the majority of participants (89%) were aged 20 ≤ 30 years, and the same percentage had 1–5 years of professional experience. Nearly half of the participants (48.5%) were female, and more than two-thirds (67.5%) held a Bachelor of Science in Nursing degree.

The findings presented in Table 1 indicate that nurses reported moderate to high levels across sustainable leadership practices, workplace integration, and resilience, with overall mean percentage scores of 72.2%, 72.3%, and 74.3%, respectively. Within sustainable leadership, ethical decision-making recorded the highest mean percentage score (87.6%), followed by environmental stewardship (81.8%) and vision and long-term thinking (80.5%). In contrast, comparatively lower scores were observed in accountability and transparency (64.3%) and innovation and adaptability (65.0%). Regarding workplace integration, teamwork and cooperation achieved the highest score (73.6%), followed closely by support and inclusion (72.7%) and adaptation and engagement (72.5%), while organizational belonging showed the lowest score (70.5%). For resilience, coping strategies ranked highest (78.7%), followed by emotional stability (74.9%) and adaptability (74.5%), whereas optimism and positive outlook recorded the lowest score (71.3%).

The Table 2 indicated generally moderate to high levels across sustainable leadership, workplace integration, and resilience. In sustainable leadership, ethical decision-making (87.6%) and vision and long-term thinking (80.5%) were the strongest areas, while accountability and transparency (64.3%) and innovation and adaptability (65.0%) were relatively lower. Overall, sustainable leadership was moderately high (72.2%).

Workplace integration showed consistent levels across all dimensions, with teamwork and cooperation recording the highest score (73.6%), and the total score was 72.3%, indicating a generally cohesive work environment.

Resilience was slightly higher overall (74.3%), with coping strategies (78.7%) and emotional stability (74.9%) being the strongest components, reflecting good adaptive capacity among participants.

Table 3 showed that all participants (100%) perceived sustainable leadership at a strong level. Regarding workplace integration, half of the participants (50%) reported a high level, while the remaining half demonstrated a moderate level. In terms of resilience, more than one quarter of participants (27.5%) demonstrated a high level of resilience, whereas 30.5% and 42.0% reported moderate and low levels, respectively.

Pearson correlation analysis presented in Table 4 demonstrated a statistically significant positive correlation between sustainable leadership and workplace integration (r = 0.43, *p* < 0.001). Sustainable leadership was also positively correlated with resilience (r = 0.24, *p* < 0.001). Also, workplace integration was positively correlated with resilience (r = 0.59, *p*< 0.001). These findings indicate that higher levels of sustainable leadership were associated with better workplace integration and greater resilience among staff nurses.

Table 5 shows that sustainable leadership was found to be a significant positive predictor of both outcomes. Specifically, sustainable leadership significantly predicted workplace integration (β = 0.43, t = 6.68, *p* < 0.001), accounting for 18% of the variance (R^2^ = 0.18, F = 44.6, *p* < 0.001). Similarly, sustainable leadership significantly predicted resilience (β = 0.24, t = 3.53, *p* < 0.001), explaining 5.8% of the variance (R^2^ = 0.058, F = 12.5, *p* < 0.001).

Table 6 presented the results of a linear regression analysis examining sustainable leadership as a predictor of resilience. The model showed that sustainable leadership was a statistically significant positive predictor of resilience (B = 0.56, β = 0.24, t = 3.53, *p* < 0.001), indicating that higher levels of sustainable leadership were associated with higher resilience scores. This suggests that sustainable leadership had a meaningful, albeit modest, influence on resilience among participants.

## 4. Discussion

1. Sample Characteristics and Methodological Context

The characteristics of the surveyed cohort indicate a sample comprising predominantly young, early-career nurses, with 89% aged 20–29 years and an identical proportion reporting 1–5 years of professional experience. Furthermore, slightly more than half of the participants identified as male (51.5%), and the majority held a Bachelor of Science in Nursing degree (67.5%), illustrating a demographic profile that is both young and academically prepared. These parameters align closely with broader international workforce trends pointing toward the increased participation of younger clinicians and a heightened institutional focus on baccalaureate nursing education to address modern healthcare demands [27]. Recent investigations emphasize that career-related and organizational variables are critical during these formative professional years, noting that structured career progression frameworks and supportive initial role experiences heavily dictate workforce retention trajectories and overall professional development [28]. Despite these baseline strengths, the high concentration of early-career individuals and the disproportionately large representation of male participants impose boundaries on the generalizability of the empirical findings. Prior comparative studies have identified statistically significant variations in psychological resilience, career socialization trajectories, and workplace perceptions across diverse age brackets and gender cohorts, which indicates that more heterogeneous population samples would likely generate distinct empirical outputs [29,30]. Consequently, the observations derived from this study necessitate cautious interpretation when extrapolated to older, highly experienced, or demographically balanced nursing populations.

2. Descriptive Analysis of Sustainable Leadership, Workplace Integration, and Resilience

The descriptive findings reveal moderate-to-high mean percentage scores across all evaluated core constructs, registering at 72.2% for sustainable leadership, 72.3% for workplace integration, and 74.3% for resilience. A detailed breakdown of the sustainable leadership dimensions demonstrates that ethical decision-making (87.6%), environmental stewardship (81.8%), and vision and long-term thinking (80.5%) constitute the primary institutional strengths, whereas accountability and transparency (64.3%) alongside innovation and adaptability (64.3% to 65.0%) represent the lowest scoring subdimensions. Within the domain of workplace integration, teamwork and cooperation (73.6%) together with support and inclusion (72.7%) achieved the highest ratings, contrasting with organizational belonging, which recorded the lowest score (70.5%). Similarly, an examination of resilience highlights coping strategies (78.7%) and emotional stability (74.9%) as leading protective subdimensions, while optimism and positive outlook (71.3%) emerged as the most vulnerable component. This configuration characterizes a professional cohort equipped with robust ethical foundations and team-level synergy, yet constrained by deficient accountability architectures, limited innovative capacity, and fragile structural belonging—an organizational profile frequently observed in post-pandemic healthcare environments.

The exceptionally high score recorded for ethical decision-making finds strong convergence across contemporary literature streams. For instance, a cross-sectional survey executed by McKenna and Jeske among 209 professional nurses demonstrated that ethical leadership serves as a positive driver of work engagement while functioning as a negative predictor of emotional exhaustion and voluntary turnover intention [31]. This is reinforced by the systematic review and meta-analysis conducted by Kim, Jeong, and Seo, encompassing 21 independent studies, which concluded that ethical leadership exerts profound positive effects on nurses’ job satisfaction, organizational commitment, and task performance [32]. Additional supporting evidence is provided by Pakizekho and Barkhordari-Sharifabad, whose descriptive-analytical research involving 230 nurses established that ethical leadership reliably predicts both professional conscientiousness and moral courage [33]. Similar positive relationships linking ethical leadership, ethical climate, and organizational citizenship behaviors were confirmed by Aloustani et al. in a cohort of 270 nurses [34], while Jandaghian-Bidgoli et al. extended these empirical associations to clinical performance metrics via a systematic review of 16 observational and qualitative studies [35].

In parallel, the robust scores observed in teamwork and cooperation align directly with the meta-review performed by Wei et al., which evaluated 36 systematic reviews and identified team-level integration as a paramount facilitator of interprofessional clinical collaboration [36]. Pomare and colleagues corroborated these dynamics in a critical review of 34 hospital-based interprofessional studies, reporting moderate-to-strong evidence linking collaborative frameworks to positive team outcomes [37]. The operational delivery of these collaborative mechanisms was further detailed in a scoping review by Djaharuddin et al., confirming that effective interprofessional integration depends heavily on mutual professional respect, shared role comprehension, and transparent communication protocols [38]. Furthermore, multinational empirical data from Shaw’s survey of 1200 healthcare workers across four distinct countries demonstrated that hospitals operating under established interprofessional collaboration structures achieve measurable operational gains, including a 20% reduction in patient readmissions and a 15% enhancement in treatment compliance [39].

Regarding the elevated coping strategies score, the findings mirror the multi-center focus group study by Cooper, Leslie, and Brown, which emphasized that individual nurses independently cultivate active coping mechanisms to navigate daily workplace adversity, though environmental determinants and direct managerial performance heavily modulate this capacity [40]. This dynamic is echoed in the comprehensive concept analysis by Cooper, Brown, and Rees, which validated coping strategies as an indispensable structural component of the psychological resilience construct within nursing populations [41].

Conversely, the depressed scores registered for accountability and transparency reflect structural vulnerabilities extensively documented in the toxic leadership literature. The absence of substantive organizational support systems, combined with opaque operational accountability and restricted access to leadership training, creates an environment conducive to dysfunctional leadership behaviors that systematically amplify fear and professional insecurity among clinical staff [42]. This is underscored by Abdelaliem and Abou Zeid, whose cross-sectional investigation of 235 nurses established a significant association between toxic leadership practices and diminished organizational performance, with employee silence serving as a key mediating variable [43]. Qualitative insights from Chesterton et al. further illustrate that clinical managers are frequently perceived by nursing personnel as remote, inaccessible, and indifferent, thereby fostering profound sentiments of professional devaluation [44].

The comparatively low scores associated with innovation and adaptability reflect systemic operational constraints rather than individual attitudinal deficits. George and Jackson observed similar structural barriers in acute care nursing evaluations, noting that translating innovative leadership requirements into routine clinical practice necessitates deliberate strategic planning and comprehensive institutional evaluation frameworks [45]. Ayan similarly argued that managerial nurses are continuously encumbered by competing operational demands that restrict the time and energy available for adaptive leadership behaviors [46].

The reduced organizational belonging scores run parallel to recent empirical findings by Murray and Chiotu involving 411 interprofessional healthcare workers, which demonstrated that while belonging represents a fundamental human motivator, clinical staff across various tenure lengths, genders, and employment statuses struggle to experience genuine organizational integration [47]. Commentary by Avery in *Nurse Leader* corroborates this trend, documenting a steady decline in healthcare worker engagement exceeding 3.7% over a three-year observation window [48]. Furthermore, Mitchell and colleagues highlighted that despite widespread recognition of diversity, equity, inclusion, and belonging (DEIB) initiatives, institutional embedding within daily clinical operations remains inconsistent and frequently falls short of target implementation benchmarks [49].

Finally, the depressed scores observed for optimism and positive outlook mirror the widespread psychological aftermath documented in post-pandemic healthcare literature. Choudhry and Sharma’s meta-analysis of 55 post-pandemic nursing studies recorded elevated rates of emotional exhaustion, depersonalization, and diminished personal accomplishment, with professional optimism suffering severe degradation under prolonged chronic workplace stress [50]. Catarelli et al. similarly reported that nurse burnout persists at moderate-to-high levels even following the acute abatement of pandemic phases, identifying optimism as one of the most heavily impacted resilience subdimensions [51]. Multi-country data from Lee et al. reinforced these observations, emphasizing that fostering positive work environments, bolstering structural empowerment, and deploying targeted resilience initiatives remain mandatory countermeasures against declining optimism among clinical staff [52].

Collectively, the convergent pattern of vulnerability spanning accountability, innovation, belonging, and optimism points toward an underlying unifying mechanism—namely, systemic institutional strain and exhausted managerial bandwidth—rather than isolated personal or departmental failures [53].

3. Distributional Analysis and Variable Stratification

The distributional categorization presented in Table 3 indicates that the entire participant cohort (100%) achieved classification within the strong category for sustainable leadership. In contrast, workplace integration demonstrated an equal partition between moderate (50%) and high (50%) performance levels. Resilience, however, exhibited substantial heterogeneity, with 42.0% (n=84) classified as low, 30.5% (n=61) as moderate, and 27.5% (n=55) as high. The concentration of nearly half of the sample within the low-resilience bracket constitutes a critical empirical indicator requiring institutional intervention, particularly given that the single largest demographic grouping falls beneath the moderate performance threshold.

This 42% prevalence of low resilience aligns closely with post-pandemic empirical trends. Nantsupawat et al. documented that even after the relaxation of acute pandemic pressures, national rates of burnout and turnover intention among 2500 U.S. nurses remained exceptionally elevated, with a substantial proportion exhibiting suppressed psychological resilience profiles [54]. Similarly, Çıtak and colleagues reported through a systematic review of 15 quantitative studies that between 35% and 45% of young clinical nurses consistently populate low-resilience tiers [55]. Tabakakis expanded upon these findings using an explanatory sequential mixed-methods design, establishing that inferior practice environments and high exposure to clinical adversity are significantly predictive of diminished psychological resilience and elevated psychological distress [56]. Furthermore, a scoping review by Budi and Eka confirmed that professional resilience is inherently multidimensional, shaped by an intricate matrix of demographic, psychological, and organizational variables that generate heterogeneous distribution patterns across healthcare settings [57].

Regarding the bimodal split observed in workplace integration, Avery’s analysis of large-scale healthcare feedback data indicated that roughly one-third of clinical workers experience operational disengagement, and that the introduction or removal of institutional DEIB frameworks can induce significant shifts in perceived integration across the workforce [58]. Mitchell et al. noted that successful DEIB integration typically demands sustained multi-year implementation cycles, often resulting in a long tail of partial workforce integration rather than immediate, uniform improvement [59].

With respect to the uniform 100% classification in sustainable leadership, methodological considerations must be acknowledged. Cooper, Brown, and Rees observed that self-report leadership instruments administered in professional environments characterized by high social desirability frequently cluster toward the upper limits of the distribution scale, suggesting that absolute uniformity reflects measurement ceiling effects alongside genuine leadership competence [60]. This observation echoes cautions raised by McKenna and Jeske regarding the susceptibility of self-rated ethical leadership metrics to inflated perception biases, prompting recommendations for multi-source performance evaluation protocols [61]. Aloustani et al. similarly emphasized that organizational ethical climate assessments require methodological triangulation beyond subjective employee perceptions to yield valid administrative inferences [62]. Moreover, bibliographic evaluations by Bulut demonstrate that psychological and leadership measurement instruments vary widely across published literature, rendering uncalibrated cross-study comparisons of categorical thresholds potentially unreliable [63]. Khaleghparast et al. further reported that self-report resilience scores frequently diverge from qualitative accounts of lived coping experiences, with quantitative instruments tending to overestimate actual resilience capacity relative to qualitative evaluations [64].

4. Correlational and Regression Dynamics

The Pearson correlation matrix detailed in Table 4 establishes a moderate positive correlation between sustainable leadership and workplace integration (r=0.425), a weak positive correlation between sustainable leadership and resilience (r=0.241), and a moderate-to-strong positive correlation between workplace integration and resilience (r=0.582). The differential magnitudes of these statistical relationships suggest that sustainable leadership influences nurse resilience primarily through workplace integration operating as a partial mediator—implying that direct leadership interventions alone will yield restricted effects on resilience unless concurrent measures are taken to strengthen team-level integration.

The identified leadership-integration correlation (r=0.425) corresponds with Olaitan’s qualitative findings from 35 healthcare executives, which emphasized that successful institutional transformation requires a deliberate equilibrium between structural and relational approaches, with interpersonal engagement acting as a core catalyst [65]. Chivaka similarly observed that nurse managers play an indispensable role in transforming fragmented clinical personnel into cohesive, high-performing operational teams [66], while Pomare et al. noted that collaborative healthcare cultures are intrinsically tied to leadership behaviors that intentionally cultivate team integration [54].

Conversely, the weaker statistical linkage observed between sustainable leadership and resilience (r=0.241) finds support in Sihvola and colleagues’ scoping review, which concluded that while nurse leaders are vital for crisis navigation, their supportive impact is largely catalytic rather than directly deterministic [67]. Förster et al. similarly identified individual situational and behavioral attributes as more proximal determinants of resilience than top-down leadership characteristics [68]. Meanwhile, the robust correlation linking workplace integration to resilience (r=0.582) aligns with Cooper, Leslie, and Brown’s multi-center research, which underscored that daily workplace conditions, organizational philosophy, managerial competence, and immediate team dynamics directly govern resilience outcomes [69]. Henshall, Davey, and Jackson reinforced this perspective, characterizing integration and resilience as fluid, continuous processes requiring reinforcement across organizational, cultural, and managerial dimensions [70].

To further test these associative patterns, the simple regression models presented in Table 5 indicate that sustainable leadership significantly predicts workplace integration ($R^{2}=0.180,F=43.42,p<0.001$), accounting for 18% of the total variance. However, sustainable leadership accounted for only 5.8% of the variance in resilience ($R^{2}=0.058,F=12.15,p<0.001$), confirming that leadership acts as a meaningful yet partial driver of integration, while its direct predictive power regarding resilience is too modest to justify single-variable intervention strategies. The 18% variance explained in integration is consistent with findings by Swen et al., who reported that inclusive leadership behaviors account for significant variance in nursing staff retentions [71]. Munir’s systematic review on transformational leadership similarly noted strong associations between leadership actions and organizational commitment, mediated primarily by interpersonal trust [69].

In contrast, the modest 5.8% variance explained in resilience aligns with Henshall, Davey, and Jackson’s conclusions that personal resilience is governed by a multitude of intersecting variables, rendering organizational interventions alone insufficient for driving large-scale psychological improvement [67]. Khaleghparast et al. and Çıtak et al. reported similar empirical observations, noting that organizational factors account for only a minor fraction of resilience variance, whereas individual psychological attributes and multifaceted environmental conditions contribute the majority of explanatory power [58,61]. Furthermore, analyses of toxic leadership, suggest an asymmetric reporting bias, wherein dysfunctional or toxic leadership behaviors account for disproportionately larger proportions of variance in negative workforce outcomes than positive leadership styles contribute to favorable resilience metrics. In contrast, evidence from psychiatric nursing settings indicates that strengthening nurses’ mindfulness can enhance well-being and leadership skills, highlighting the potential value of positive, nurse-focused interventions in promoting healthier leadership outcomes [44,67].

Finally, the regression model detailed in Table 6 demonstrates a statistically significant positive association between sustainable leadership and resilience (β=0.56,t=3.48,p<0.001, constant = 24.45), indicating that each incremental unit increase in sustainable leadership corresponds to a 0.56-unit elevation in resilience. While consistent with prior literature linking supportive and ethical governance practices to staff adaptation [42,43,49,64,71], the modest overall effect size underscores that a substantial proportion of resilience variance remains unexplained. Consequently, future empirical investigations must incorporate expanded multivariate frameworks, control for demographic and organizational confounders, and rigorously test structural equation models featuring workplace integration as an active mediating mechanism.

5. Study Limitations

Several limitations should be considered when interpreting the findings. First, the cross-sectional correlational design precludes causal inferences between sustainable leadership, workplace integration, and resilience. Second, data were collected using self-reported questionnaires, which may have introduced response bias and common method bias. Third, although the study achieved a relatively high response rate (83.3%), participants were recruited using convenience sampling from a single tertiary healthcare institution. However, this methodological approach warrants careful consideration, as convenience sampling from a single site may introduce selection bias and restrict the representativeness of the sample. Consequently, the findings should be generalized with caution to nurses working in other healthcare settings or organizational contexts. In addition, resilience and workplace integration are multidimensional constructs that may be influenced by factors not examined in the present study, such as organizational culture, staffing adequacy, workload, psychological resources, and organizational climate. Furthermore, the regression analyses included sustainable leadership as the sole independent variable and did not adjust for potential demographic, professional, or organizational confounders (e.g., age, educational level, years of experience, and workload). The modest proportion of explained variance suggests that additional factors contribute to workplace integration and resilience.

Despite these limitations, this study contributes to the growing body of evidence on sustainable leadership in healthcare settings by demonstrating its association with workplace integration and resilience among staff nurses. Future research should employ longitudinal and multicenter designs with probability sampling and multivariable analyses to improve causal inference, reduce selection bias, and enhance the generalizability of the findings.

## 5. Conclusions

The findings of this study indicate that sustainable leadership is positively associated with workplace integration and resilience among staff nurses. Nurses who perceived higher levels of sustainable leadership also reported better workplace integration and greater resilience. These findings support the growing recognition of leadership as an important organizational factor associated with workforce adaptation and well-being in healthcare settings.

The study further demonstrated that sustainable leadership showed a stronger association with workplace integration than with resilience. Although leadership was significantly associated with both outcomes, the relatively modest explained variance suggests that workplace integration and resilience are influenced by multiple organizational and individual factors beyond leadership alone.

Overall, the findings suggest that sustainable leadership may be an important organizational factor associated with more supportive, collaborative, and adaptive healthcare work environments. However, given the cross-sectional design and single-site convenience sample, these findings should be interpreted with appropriate caution and may not be generalizable to all healthcare settings. Future multicenter longitudinal studies are warranted to confirm these associations and further explore additional factors influencing workplace integration and resilience among nurses.

## 6. Recommendations

Recommendations for Practice

Healthcare organizations should incorporate sustainable leadership principles into leadership development and managerial training programs, with emphasis on ethical decision-making, staff empowerment, accountability, and social responsibility.

Nurse managers should implement strategies that promote workplace integration, including structured orientation programs, mentorship, team collaboration, and participative decision-making.

Healthcare institutions should establish supportive work environments that enhance nurses’ psychological well-being and resilience, particularly in high-stress clinical settings.

Organizational interventions such as resilience training, peer-support initiatives, and workload management programs should be integrated alongside leadership development efforts.

Recommendations for Research

Future studies should employ longitudinal or experimental designs to better examine potential causal relationships between sustainable leadership and workforce outcomes.

Researchers are encouraged to investigate additional factors that may influence workplace integration and resilience, including organizational culture, staffing adequacy, psychological capital, emotional intelligence, and workload burden.

Further studies across different healthcare sectors and cultural contexts are recommended to improve the generalizability of findings and identify contextual influences on sustainable leadership practices.

Recommendations for Policy

Healthcare policymakers and administrators should consider sustainable leadership competencies when selecting and evaluating nursing leaders.

National and institutional frameworks supporting sustainable leadership practices should be developed to promote workforce sustainability, resilience, and quality healthcare delivery.

Policies supporting staff well-being, professional development, and healthy work environments should be integrated into healthcare organizational strategies.

## Figures and Tables

**Table 1:** Demographic characteristics of the study participants (n = 200).

Variable	Category	n	%
Age (years)	20–<30	178	89.0
	30–<40	22	11.0
	Range Mean ± SD	20–39 25.72 ± 3.09	
Years of experience	1–5	178	89.0
	>10	22	11.0
	Range Mean ± SD	1–12 2.80 ± 1.22	
Gender	Female	97	48.5
	Male	103	51.5
Educational qualification	Associate technical degree	26	13.0
	Bachelor of Science in Nursing	135	67.5
	Master’s degree in Nursing	39	19.5

**Table 2:** Descriptive statistics of sustainable leadership, workplace integration, and resilience dimensions.

Variable/Dimension	Min	Max	Mean	SD	Mean %
Sustainable Leadership					
Vision and long-term thinking	5	10	8.05	1.57	80.5
Ethical decision-making	8	10	8.76	0.90	87.6
Stakeholder engagement	2	10	6.74	2.39	67.4
Social responsibility	2	10	6.61	2.49	66.1
Environmental stewardship	4	8	6.54	1.06	81.8
Innovation and adaptability	2	10	6.50	2.29	65.0
Accountability and transparency	2	10	6.43	2.09	64.3
Capacity building	2	10	6.68	2.31	66.8
Total sustainable leadership	27	78	56.29	15.10	72.2
Workplace Integration					
Communication and collaboration	4	20	14.45	3.87	72.2
Teamwork and cooperation	3	15	11.04	2.92	73.6
Organizational belonging	3	15	10.57	3.03	70.5
Support and inclusion	7	15	10.90	2.52	72.7
Adaptation and engagement	7	15	10.88	3.16	72.5
Total workplace integration	24	80	57.83	15.51	72.3
Resilience					
Emotional stability	7	15	11.24	2.58	74.9
Adaptability	8	15	11.17	2.66	74.5
Coping strategies	9	15	11.80	2.13	78.7
Optimism and positive outlook	6	15	10.69	2.62	71.3
Professional endurance	6	15	10.82	2.53	72.1
Total resilience	36	75	55.72	12.53	74.3

**Table 3:** Distribution of participants according to levels of study variables.

Variable	Level	N	%
Sustainable leadership	Strong	200	100.0
Workplace integration	Moderate	100	50.0
	High	100	50.0
Resilience	Low	84	42.0
	Moderate	61	30.5
	High	55	27.5

**Table 4:** Correlation between sustainable leadership, workplace integration, and resilience.

Variables	Total Sustainable Leadership	Total Workplace Integration
Total workplace integration	r = 0.43	1
Total resilience	r = 0.24	r = 0.59

**Table 5:** Linear regression analysis of the effect of sustainable leadership on workplace integration and resilience.

Dependent Variable	Predictor	B	SE	β	T	*p*	R^2^	F	*p* (Model)
Workplace integration	Constant	−1.54	8.92	—	−0.17	0.865	0.18	44.6	< 0.001
	Total sustainable leadership	1.06	0.16	0.43	6.68	< 0.001			
Resilience	Constant	24.45	8.88	—	2.75	0.006	0.058	12.5	< 0.001
	Total sustainable leadership	0.56	0.16	0.24	3.53	< 0.001			

Note: B = unstandardized coefficient; SE = standard error; β = standardized coefficient.

**Table 6:** Linear regression analysis predicting resilience from sustainable leadership.

Predictor	B	SE	Β	t	*p*
Constant	24.45	8.88	—	2.75	0.01
Total sustainable leadership	0.56	0.16	0.24	3.53	<0.001

## Data Availability

The data presented in this study are available on reasonable request from the corresponding author due to privacy and ethical restrictions.
